# System-Level Factors and Time Spent on Electronic Health Records by Primary Care Physicians

**DOI:** 10.1001/jamanetworkopen.2023.44713

**Published:** 2023-11-22

**Authors:** Lisa S. Rotenstein, A. Jay Holmgren, Daniel M. Horn, Stuart Lipsitz, Russell Phillips, Richard Gitomer, David W. Bates

**Affiliations:** 1 Brigham and Women’s Hospital, Boston, Massachusetts; 2 Harvard Medical School, Boston, Massachusetts; 3 University of California at San Francisco; 4 Massachusetts General Hospital, Boston; 5 Harvard Center for Primary Care, Boston, Massachusetts

## Abstract

**Importance:**

Primary care physicians (PCPs) spend the most time on the electronic health record (EHR) of any specialty. Thus, it is critical to understand what factors contribute to varying levels of PCP time spent on EHRs.

**Objective:**

To characterize variation in EHR time across PCPs and primary care clinics, and to describe how specific PCP, patient panel, clinic, and team collaboration factors are associated with PCPs’ time spent on EHRs.

**Design, Setting, and Participants:**

This cross-sectional study included 307 PCPs practicing across 31 primary care clinics at Massachusetts General Hospital and Brigham and Women’s Hospital during 2021. Data were analyzed from October 2022 to October 2023.

**Main Outcomes and Measures:**

Total per-visit EHR time, total per-visit pajama time (ie, time spent on the EHR between 5:30 pm to 7:00 am and on weekends), and total per-visit time on the electronic inbox as measured by activity log data derived from an EHR database.

**Results:**

The sample included 307 PCPs (183 [59.6%] female). On a per-visit basis, PCPs spent a median (IQR) of 36.2 (28.9-45.7) total minutes on the EHR, 6.2 (3.1-11.5) minutes of pajama time, and 7.8 (5.5-10.7) minutes on the electronic inbox. When comparing PCP time expenditure by clinic, median (IQR) total EHR time, median (IQR) pajama time, and median (IQR) electronic inbox time ranged from 23.5 (20.7-53.1) to 47.9 (30.6-70.7) minutes per visit, 1.7 (0.7-10.5) to 13.1 (7.7-28.2) minutes per visit, and 4.7 (4.1-5.2) to 10.8 (8.9-15.2) minutes per visit, respectively. In a multivariable model with an outcome of total per-visit EHR time per visit, an above median percentage of teamwork on orders was associated with 3.81 (95% CI, 0.49-7.13) minutes per visit fewer and having a clinic pharmacy technician was associated with 7.87 (95% CI, 2.03-13.72) minutes per visit fewer. Practicing in a community health center was associated with fewer minutes of total EHR time per visit (5.40 [95% CI, 0.06-10.74] minutes).

**Conclusions and Relevance:**

There is substantial variation in EHR time among individual PCPs and PCPs within clinics. Organization-level factors, such as team collaboration on orders, support for medication refill functions, and practicing in a community health center, are associated with lower EHR time for PCPs. These findings highlight the importance of addressing EHR burden at a systems level.

## Key Points

**Question:**

How are specific primary care physician (PCP), patient panel, clinic, and team collaboration factors associated with PCPs’ electronic health record (EHR) time?

**Findings:**

In this cross-sectional study of 307 PCPs across 31 primary care practices at Massachusetts General Hospital and Brigham and Women’s Hospital during 2021, organization-level factors, such as greater team collaboration on orders, the presence of specific clinic staff, and practicing in a community health center, were associated with significantly lower per-visit EHR time across multiple categories.

**Meaning:**

These findings suggest the importance of addressing EHR burden at a systems level.

## Teaser

This cross-sectional study assesses the time primary care physicians spent on the electronic health record in 31 primary care clinics in 2 academic medical center–based primary care networks in 2021.

## Introduction

Primary care physicians (PCPs) spend the most time on the EHR of any specialty.^1^ While more time on the EHR is associated with some better preventive care outcomes,^2^ those gains may come at a cost, as physician time spent on the EHR, particularly after hours, is associated with emotional exhaustion and higher rates of burnout.^3,4^ Understanding what factors shape PCPs’ use of EHRs and contribute to varying EHR time is a critical goal for both health systems and policy makers navigating how to deliver high quality care without overburdening the clinical workforce.

There is known variation in PCPs’ time on the EHR and work across multiple dimensions. For example, there is variation in time spent across different primary care specialties^5^ and among physicians within primary care specialties.^6^ Female physicians spend more time on EHRs^7^ and receive more electronic inbox messages from both patients and staff.^8^ Additionally, clinic-level factors, such as support for documentation and patient panel factors (eg, medical complexity or social characteristics), may influence time spent on the EHR. Among PCPs, use of scribes has been associated with less self-reported EHR time^9^ and with increased after-hours record completion.^10^

While there is increasing evidence regarding how single factors, such as physician sex or specialty, scribe use, or specific panel characteristics, are associated with EHR time variation, there is limited understanding of how the combination of PCP, panel, clinic, and team collaboration factors are associated with EHR time. A greater understanding of the confluence of factors associated with differing time expenditure could inform clinic and panel design as well as investment in resources for EHR and workplace experience optimization. For example, scribes may not be as impactful in reducing EHR time expenditure in a clinic with a high ratio of support staff to physicians as in clinics without these additional supports. It is therefore critical to investigate how PCP, team, and clinic-level factors are associated with EHR time burden for PCPs.

Given this context, we sought to answer 3 questions. First, how does PCP time spent on EHR (in total, during pajama time [defined as any active time between the hours of 5:30 PM and 7:00 AM], and on the electronic inbox) vary across PCPs and primary care clinics in a large health system? Second, how does the presence of primary care clinic resources and team collaboration vary across PCPs and clinics? Third, what PCP, patient panel, clinic, and team collaboration factors are associated with time spent on the EHR by PCPs?

## Methods

This cross-sectional study followed the Strengthening the Reporting of Observational Studies in Epidemiology (STROBE) reporting guideline. Given its focus on secondary data analysis, this analysis was considered exempt by the institutional review board of Mass General Brigham and informed consent was waived.

### Sample

Our sample included all 338 practicing attending PCPs at Brigham and Women’s Hospital (BWH) and Massachusetts General Hospital (MGH) (eAppendix 1 in Supplement 1). These academic medical centers, which have separate primary care networks, are both part of the Mass General Brigham system, implemented the same instance of the same EHR vendor system (Epic), and use similar panel design and time allocation structures. Their clinics are in both urban and suburban sites and include 6 community health centers (CHCs). We focus our analyses on the 316 attending PCPs who care for specified patients longitudinally since some aspects of EHR time expenditure, such as time spent on the electronic inbox, differ by whether PCPs see patients over time. Resident PCPs were not included in this analysis.

For PCPs with a patient panel for all of 2021, we extracted data regarding their personal demographics, patient panels, and productivity; their clinic site’s characteristics; information regarding their team collaboration; and EHR use data. We excluded 9 PCPs who entered practice in 2021 and did not have a full year of data. Our final analytic sample was 307 PCPs.

### Potential Contributors to EHR Time

#### PCP Factors

For each PCP, we received BWH and MGH administrative record data regarding their sex (male or female), the proportion of their full time equivalent (FTE) that they practiced clinical medicine in the primary care clinic setting (hereafter referred to as clinical FTE), and the number of years since their completion of residency. We additionally identified the primary care clinic site where each PCP spent most of their clinical time.

#### Panel Factors

For each PCP’s panel, we collected information about their average panel risk score (based on a US Health and Human Services–derived risk score [average score 1.0, with healthier and less healthy patients with scores below and above 1.0, respectively]^12^ for adults with commercial insurance that incorporates information about age, sex, and hierarchical condition categories),^11^ and the percentage of female patients and patients with Medicaid insurance on their panel in 2021. In the Mass General Brigham system, PCP attribution is determined by the name in the PCP field in the EHR for each patient.

#### Team Collaboration Factors

We collected data regarding 2 team collaboration factors: (1) whether each PCP used a scribe in 2021 and (2) the percentage of orders placed by a physician that have a team contribution, such as a nonphysician member of the care team pending the order for PCP signature. Data regarding scribe use was derived from MGH and BWH administrative records. The Epic Signal database, whose features and available measures are described in the EHR Use Data section, additionally provides data on the percentage of orders placed by a physician that have a team contribution, and we calculated mean values for this metric for each PCP throughout the 12-month period. Hereafter, we refer to this metric, which is measured on a scale of 0 to 100 percentage points, as team contribution to orders.

#### Productivity Characteristics

Using the EHR database data logs, we quantified the total number of electronic inbox messages each PCP received during the 2021 calendar year and the total number of appointments (hereafter referred to as visits; all scheduled for 30 minutes) conducted by each PCP during 2021. This included both in-person and telehealth visits; however, our available data sources did not differentiate between these visit types. Visits primarily provided by a resident physician were not included in this analysis.

#### Primary Care Clinic Factors

We collected information regarding each primary care clinic’s medical assistant (MA) FTE to clinician (physician or advanced practice clinician) FTE ratio. We also collected the nurse FTE to clinician FTE ratio, secretarial staff FTE to clinician FTE ratio, whether practice has a pharmacy technician, and whether the clinic is a CHC.

### EHR Use Data

We extracted EHR use data for January to December 2021 for all PCPs from the EHR database’s metadata platform, Epic Signal. We then summed values from each of the 12-monthly EHR use data extracts and normalized by total yearly visits to calculate physician-level per visit means of the following EHR use metrics: total EHR time, pajama time (defined as any active time between the hours of 5:30 pm and 7:00 am), and time spent in the electronic inbox (the EHR database module for sending and receiving patient, staff, and system messages). We secondarily extracted data on time spent outside of scheduled clinical hours, which measures time during clinical sessions plus 30 minutes before and after a session. All EHR system time metrics are based on the time during which a user is performing active tasks. If no activity is detected for 5 seconds, the system stops counting time.^13^

### Analyses

We first descriptively analyzed distributions of PCPs’ individual, patient panel, team collaboration, and workload characteristics. Additionally, we analyzed the distributions of staffing ratios by clinic site. We then characterized the distribution of median total per-visit EHR time, per-visit pajama time, and per-visit electronic inbox time across individual PCPs and across PCPs grouped by clinic site.

After characterizing the univariate associations between PCP, panel, clinic, and team collaboration factors, we used generalized estimating equations (identity link and normal distribution) with standard errors clustered by clinic to determine the association of PCP, patient panel, team collaboration, and primary care clinic site factors with total per-visit EHR time, per-visit pajama time, and per-visit electronic inbox time. PCP factors included sex, years since residency, and clinical FTE. Patient panel factors included a binary variable for above vs below sample median panel risk score, a binary variable for above vs below sample median percentage of panel with Medicaid primary insurance, and a binary variable for above vs below sample median percentage of female patients on panel. Team collaboration factors included a binary variable for whether a PCP used a scribe and a binary variable for above vs below sample median percentage teamwork on orders. Primary care clinic site factors included a binary variable for medical assistant (MA) FTE to clinician (physician or advanced practice clinician) FTE ratio above or below sample median; a binary variable for nurse FTE to clinician FTE ratio above or below sample median; a binary variable for secretarial staff FTE to clinician FTE ratio above or below sample median; a binary variable for whether the clinic has a pharmacy technician; and whether the clinic is a CHC. We additionally adjusted for the institution the PCP was associated with (given potential cultural differences in the institutions that could influence EHR time), each PCP’s panel size (given some panel sizes disproportionate to PCPs’ clinical FTE, and each PCP’s electronic inbox message volume (given variation beyond that explained by factors already measured). In sensitivity analyses, we characterized the distribution of time outside of scheduled hours across PCPs and PCPs grouped by clinic and constructed a multivariable model with an outcome of time outside of scheduled hours.

In further sensitivity analyses, we specified our main models with outcomes of total EHR time per visit, pajama time per visit, and electronic inbox time per visit via ordinary least squares regression (with assessment of model fit and inclusion of collinearity diagnosis). While our base models specified certain patient panel, clinic, and team collaboration variables as binary to enhance interpretability of results, in additional sensitivity analyses, we specified models via generalized estimating equations with certain panel, clinic, and team collaboration factors characterized as continuous variables or variables segmented by quartiles rather than as dichotomous variables.

All analyses were completed in SAS On Demand for Academics, 2023 (SAS Institute). A 2-sided threshold of *P* = .05 was used to determine significance. Data were analyzed from October 2022 to October 2023.

## Results

### Sample Descriptive Characteristics

Our sample included 307 PCPs, of whom 183 (59.6%) were female. As shown in Table 1, the median FTE (IQR) of PCPs in the sample was 0.5 (0.38-0.75). PCPs were a median (IQR) of 20 (9-28) years from residency completion. PCPs’ median (IQR) panel size was 837 (481-1158) patients and median (IQR) panel risk score was 1.9 (1.5-2.3). The median (IQR) percentage of each PCP’s panel comprised of female and Medicaid patients were 67.0% (37.0%-78.0%) and 10.6% (5.9%-23.0%), respectively. PCPs had completed a median (IQR) of 1082 (669-1608) visits during calendar year 2021 and received a median (IQR) of 12 445 (8164-15 771) electronic inbox messages during the year. They had a median (IQR) of 275 (249-305) days with any EHR activity during 2021.

**Table 1.** Distribution of PCP and Physician-Associated Characteristics

Characteristics	Median (IQR)
Individual PCP	
Institution, No. (%)	
BWH	139 (45.3)
MGH	168 (54.7)
Sex, No. (%)	
Female	183 (59.6)
Male	124 (40.4)
Clinical full time equivalent	0.5 (0.375-0.75)
Years postresidency	20 (9-28)
Patient panel	
No. of patients on PCP’s panel	837 (481-1158)
Panel risk score	1.9 (1.6-2.3)
Panel comprised of female patients, %	67.0 (37.0-78.0)
Panel with Medicaid Insurance, %	10.6 (5.9-23.0)
Team collaboration characteristics	
Orders with team contribution, %	4.7 (2.5-7.8)
Uses a scribe, No. (%)	83 (27.0)
Workload	
Total yearly messages	12 445 (8164-15 771)
Total yearly visits	1082 (669-1608)
Total days per year with EHR activity	275 (249-305)

Abbreviations: BWH, Brigham and Women’s Hospital; EHR, electronic health record; MGH, Massachusetts General Hospital; PCP, primary care physician.

### Team Collaboration for EHR Work

More than a quarter of physicians used a scribe during 2021 (83 physicians [27.0%]) (Table 1). A median (IQR) of 4.7% (2.5%-7.8%) of PCPs’ orders had a contribution from a nonphysician member of the clinical team.

### Distribution of EHR Time Across PCPs

**Figure 1.**
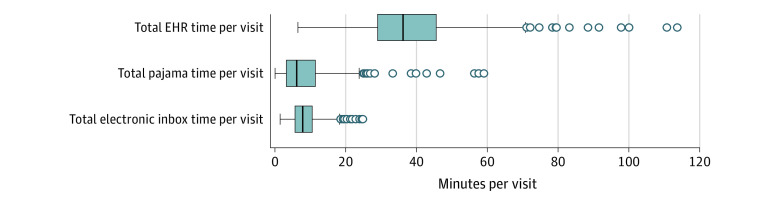
Distribution of PCPs’ Time Spent on Electronic Health Record (EHR) per Visit Lines within boxes indicate medians, boxes indicate IQRs, whiskers denote 1.5 IQR of the lower and higher quartiles, and circles denote outliers.

There was substantial variation in EHR time per visit across the PCPs in our sample (Figure 1). PCPs spent a median (IQR) of 36.2 (28.9-45.7) minutes in total on the EHR per visit, 6.2 (3.1-11.5) minutes of pajama time per visit (with similar variation observed for the outcome of time outside of scheduled hours (eAppendix 2 in Supplement 1), and 7.8 (5.5-10.7) minutes on the electronic inbox per visit.

### Distribution of EHR Time Across Clinics

**Figure 2.**
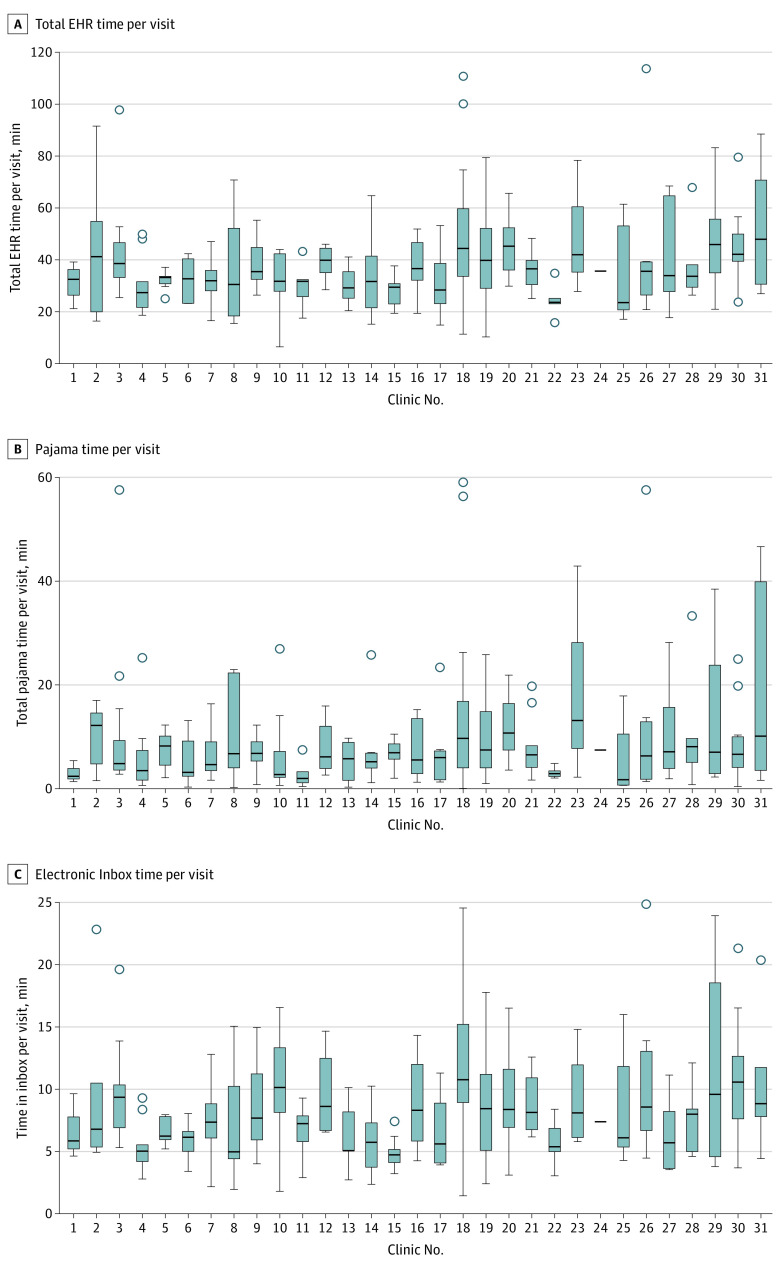
Distribution of Electronic Health Record (EHR) Time per Visit by Clinic Lines within boxes indicate medians, boxes indicate IQRs, whiskers denote 1.5 IQR of the lower and higher quartiles, and circles denote outliers.

There was also substantial variation in per-visit EHR time across the clinics in our sample (Figure 2). Median (IQR) per-visit total EHR time ranged from 23.5 (20.7-53.1) to 47.9 (30.6-70.7) minutes per visit across clinics (Figure 2). Median (IQR) per-visit pajama time ranged from 1.7 (0.7-10.5) to 13.1 (7.7-28.2) minutes across clinics (Figure 2), with similar variation observed for the outcome of time outside of scheduled hours (eAppendix 2 in Supplement 1). Finally, median (IQR) per-visit electronic inbox time ranged from 4.7 (4.1-5.2) to 10.8 (8.9-15.2) minutes across clinics (Figure 2).

### Clinic Characteristics

As shown in Table 2, of the 31 clinics in our sample, 9 (31.0%) were considered CHCs and 7 (22.5%) had a pharmacy technician. Clinics had a median (IQR) of 8 (6-10) physicians. Their median (IQR) medical assistant FTE to clinician FTE ratio was 0.72 (0.60-0.82) while their median (IQR) nurse FTE to clinician FTE ratio was 0.49 (0.35-0.55) and median (IQR) secretarial staff FTE to clinician FTE ratio was 0.87 (0.69-1.01).

**Table 2.** Distribution of Characteristics by Primary Care Clinic

Clinic-level variables	No. (%)
Institution	
BWH	15 (48.3)
MGH	16 (51.6)
Clinic has a pharmacy technician	7 (22.5)
Clinic is a community health center	9 (31.0)
No. of physicians, median (IQR)	8 (6-10)
MA FTE to clinician FTE ratio, median (IQR)	0.72 (0.60-0.82)
Nurse FTE to clinician FTE ratio, median (IQR)	0.49 (0.35-0.55)
Secretarial staff FTE to clinician FTE ratio, median (IQR)	0.87 (0.69-1.01)

Abbreviations: BWH, Brigham and Women’s Hospital; FTE, full-time equivalent; MA, medical assistant; MGH, Massachusetts General Hospital.

### Factors Associated With PCPs’ EHR Time

Univariate correlations between PCP, panel, clinic, and team collaboration factors are displayed in eAppendix 3 in Supplement 1. As shown in Table 3, in a multivariable linear regression model with an outcome of total per-visit EHR time per visit, factors associated with lesser EHR time per visit included presence of a pharmacy technician (−7.87 [95% CI, −13.72 to −2.03] minutes; *P* = .01), practicing in a CHC (−5.40 [95% CI, −10.74 to −0.06] minutes; *P* = .05), and above median team contribution to orders (−3.81 [95% CI, −7.13 to −0.49] minutes; *P* = .02). Scribe use was in the direction of lesser EHR time per visit but did not reach statistical significance (−3.32 [95% CI, −6.73 to 0.09] minutes; *P* = .06). Each additional year postresidency was associated with 0.13 (95% CI, 0.01 to 0.25) more minutes per visit (*P* = .03).

**Table 3.** Generalized Estimating Equation Models Depicting Adjusted Associations of PCP, Panel, Clinic, and Team Collaboration Factors With Total EHR Time per Visit, Total Pajama Time per Visit, and Total Electronic Inbox Time per Visit^a^

Parameter	Total EHR time per visit		Total pajama time per visit		Total inbox time per visit	
	Estimate (95% CI), min/d	*P* value	Estimate (95% CI), min/d	*P* value	Estimate (95% CI), min/d	*P* value
PCP factors						
PCP sex (female vs male)	4.20 (−1.89 to 10.29)	.18	2.56 (−0.65 to 5.77)	.12	1.03 (−0.82 to 2.88)	.27
Clinical FTE	−8.35 (−24.09 to 7.38)	.30	−5.50 (−13.52 to 2.52)	.18	−6.40 (−8.87 to −3.92)	<.001
No. of years postresidency	0.13 (0.01 to 0.25)	.03	0.15 (0.07 to 0.23)	<.001	0.00 (−0.03 to 0.03)	.94
Patient panel factors						
Above median percentage female patients on panel (yes vs no)	−1.55 (−7.76 to 4.65)	.62	−2.43 (−6.02 to 1.16)	.18	−0.34 (−1.96 to 1.28)	.68
Above median percentage patients with Medicaid on panel (yes vs no)	1.64 (−1.81 to 5.09)	.35	−0.28 (−2.36 to 1.81)	.79	−0.01 (−0.67 to 0.65)	.98
Above median panel risk score (yes vs no)	−2.24 (−6.02 to 1.53)	.24	−1.80 (−3.85 to 0.24)	.08	−0.24 (−1.25 to 0.77)	.64
Primary care clinic factors						
MA FTE to clinician FTE ratio above median (yes vs no)	−2.80 (−6.22 to 0.63)	.11	−1.02 (−2.76 to 0.72)	.25	0.33 (−0.35 to 1.00)	.34
Nurse FTE to clinician FTE ratio above median (yes vs no)	−1.74 (−4.68 to 1.19)	.24	−1.24 (−2.63 to 0.15)	.08	−0.09 (−0.78 to 0.60)	.80
Secretarial staff FTE to clinician FTE ratio above median (yes vs no)	1.76 (−1.34 to 4.87)	.27	1.65(−0.03 to 3.33)	.05	0.01 (−0.73 to 0.75)	.98
Presence of a pharmacy technician (yes vs no)	−7.87 (−13.72 to −2.03)	.01	−3.73 (−7.27 to −0.18)	.04	−1.65 (−3.05 to −0.25)	.02
Community health center (yes vs no)	−5.40 (−10.74 to −0.06)	.047	−1.97 (−4.33 to 0.40)	.10	−1.68 (−2.95 to −0.41)	.01
Team collaboration						
Use of a scribe (yes vs no)	−3.32 (−6.73 to 0.09)	.06	−0.09 (−2.01 to 1.82)	.93	−0.11 (−1.05 to 0.82)	.81
Above median team contribution to orders (yes vs no)	−3.81 (−7.13 to −0.49)	.02	−2.55 (−4.41 to −0.68)	.01	−1.48 (−2.15 to −0.80)	<.001

Abbreviations: EHR, electronic health record; FTE, full-time equivalent; MA, medical assistant; PCP, primary care physician.

^a^ Models additionally control for institution (Brigham and Women’s Hospital vs Massachusetts General Hospital), panel size (number of patients), and yearly message quantity (number of messages).

We observed similar patterns for factors associated with per-visit pajama time, time outside of scheduled hours, and per-visit electronic inbox time in multivariable models (Table 3). In a model with an outcome of pajama time per visit, having an above median team contribution to orders (−2.55 [95% CI, −4.41 to −0.68] minutes; *P* = .01) and the presence of a pharmacy technician in the clinic (−3.73 [95% CI, −7.27 to −0.18] minutes; *P* = .04) were significantly associated with lesser time. Each additional year postresidency was associated with 0.15 (95% CI, 0.07 to 0.23) additional minutes of pajama time per visit (*P* < .001). Similar trends were seen in a model with an outcome of time outside of scheduled hours (eAppendix 2 in Supplement 1). Finally, in a model with an outcome of electronic inbox time per visit, the following variables were associated with less EHR time per visit: presence of a pharmacy technician (−1.65 [95% CI, −3.05 to −0.25] minutes; *P* = .02), practicing in a CHC (−1.68 [95% CI, −2.95 to −0.41] minutes; *P* = .01), and above median team contribution to orders (−1.48 [95% CI, −2.15 to −0.80] minutes; *P* < .001).

Models specified via ordinary least squares regression yielded similar results and consistently had variance inflation factors less than 10, minimizing concern for collinearity (eAppendix 4 in Supplement 1). Models specified as generalized estimating equations with certain panel, clinic, and team collaboration factors characterized as variables segmented by quartiles (eAppendix 5 in Supplement 1) or as continuous variables (eAppendix 6 in Supplement 1) also yielded directionally consistent results.

## Discussion

In this cross-sectional study of EHR use by 307 PCPs practicing in 2 academic-medical center based primary care networks, we found substantial variation in the time spent on the EHR among individual PCPs and PCPs within clinics. Additionally, we identified factors suggestive of active teamwork on the EHR, such as team collaboration on orders and pharmacist technician staffing, as being associated with lower EHR time across multiple categories. Practicing in a CHC was also associated with lower total EHR time expenditure and time on the electronic inbox. While prior studies have described variation in EHR time across specialty types,^1^ within primary care specialties,^5^ and among physicians in the same specialty,^6^ we uniquely quantified how EHR time varies not only across physicians, but across clinical sites within a health system. Additionally, we assessed the factors contributing to variation in PCPs’ time expenditure on EHR at multiple levels, from the individual physician level to the patient panel, clinic, and team collaboration level.

Our findings provide actionable insight into how to modify primary care clinic workflows and staffing to optimize PCPs’ interactions with EHRs. Using detailed EHR action log data, we found that higher levels of team contribution to orders were associated with significantly lower total EHR time, pajama time, and time on the electronic inbox per visit. These associations were present even while controlling for the presence of staffing ratios. Prior studies have described how team-based primary care workflows, ranging from team-based documentation to team visits, enhance both clinical outcomes and experiences of care provision for physicians and other team members.^14,15^ Our findings emphasize that processes that enhance the contributions of other team members to EHR-based workflows may be particularly beneficial for optimizing EHR time.

Our findings also shed light on potential areas for investment by leaders seeking to enhance EHR-related staff support. They suggest that the presence of pharmacy technicians, who pend medication refills, troubleshoot medication fill issues, and assist physicians with prior authorizations, could help reduce EHR time in multiple categories. Although the association of scribes with lesser total EHR time did not reach statistical significance, the coefficient for this variable in our total EHR time model supports existing literature suggesting a positive effect of scribes on total EHR time expenditure.^16,17^ Prior studies have suggested that physicians who adopt scribes may be less efficient in their baseline documentation practices,^10^ limiting the ability to assess impact in cross-sectional analyses. Alternatively, it is possible that time freed by scribe use is shifted to other EHR-related tasks, obscuring an effect on overall EHR time expenditure and minimizing effects of scribe use on pajama time or time outside of scheduled hours. Of note, while the association of medical assistant to clinician FTE ratio with total EHR time per visit did not reach statistical significance, our models suggest a directionally negative association between these variables, which should be explored in larger studies.

Despite the evidence generated regarding associations between modifiable clinic and team collaboration factors associated with EHR time variation, there was still substantial variation in time expenditure at the PCP level and unexplained variation in all EHR time models. Thus, there is likely still some role for individually targeted interventions to reduce PCP-level variation in EHR time alongside system-level interventions.

We also found that practicing in a CHC was associated with less total EHR time and electronic inbox time. It is possible that this finding reflects known, lesser digital engagement among patients cared for in the community health center setting, as reflected via lower average patient medical advice request quantity per empaneled patients in our CHCs. Recent studies have demonstrated language, digital comfort, and other barriers to engagement with patient portals among populations cared for in community health and safety net settings, including those whose primary language is not English.^18,19,20^ Thus, our findings point toward continued opportunities to engage diverse populations in the primary care setting via digital means, while balancing the goal of reducing EHR burden for physicians.

On the individual PCP level, our results both shed light on new associations and extend past evidence. For example, we identified a significant, positive association of years since residency with total EHR time and pajama time per visit. Among multiple possible explanations, this association could be due to PCPs with more years of experience conducting longer visits with patients they know well or differences in PCPs’ facility with technology. Meanwhile, in contrast to other studies,^7,21^ our adjusted models did not demonstrate an association between PCP sex and our main EHR time outcomes despite associations in univariate analyses. Consistent with literature showing differences in clinic and EHR-related resource availability for female physicians^22^ and patients’ interactions with female physicians,^23,24^ our results suggest that sex differences in EHR time may be influenced by panel, clinic, or team collaboration factors rather than being solely the result of PCP sex.

### Limitations

This study has limitations. The study was based on the EHR use patterns, clinic staffing patterns, and patient panel characteristics of 2 academic medical center primary care networks, where few of the physicians practice clinical medicine full-time. Thus, the findings may not be readily generalizable to nonacademic settings, settings in which physicians devote a greater portion of their professional effort to clinical care, or outside of primary care. Given that the EHR database counts only the time that a user is actively interacting with the EHR, and categorizes work based on the screen with which a user is interacting, it likely an underestimate of the time spent by an end user. Although sufficient data was not available for this study to consider the association of percentage of patients with a primary language other than English on each PCPs’ panel with EHR time expenditure, this would be valuable to explore in future studies. Additionally, our data source did not differentiate between visits conducted in-person vs telehealth, which may be associated with differential EHR time expenditure. Finally, the cross-sectional nature of our study precludes drawing causal conclusions. Nevertheless, we believe this is the most granular study to date of the relative contribution of diverse factors to EHR time expenditure and provides potentially actionable guidance on future directions for modulating PCPs’ time spent on EHR.

## Conclusions

EHR time burden, and the burnout associated with this burden, represent a serious threat to the PCP workforce. Our study identified significant variation in EHR time across both individual PCPs and PCPs within clinics. We found that team and clinic factors, such as teamwork on orders, having a pharmacy technician, and practicing in a CHC, were associated with lesser EHR time. These findings can guide health system leaders as they develop new approaches to care delivery that address the burden of the EHR for PCPs and enhance the sustainability of modern primary care practice.

## Notes

Supplement 1. **eAppendix 1.** Flow Diagram of PCP Inclusion in Study Sample **eAppendix 2.** PCP and Clinic Variation in Time Outside of Scheduled Hours Per Visit and Factors Associated with Variation in Time Outside of Scheduled Hours Per Visit in Multivariable Generalized Estimating Equation Model **eAppendix 3.** Correlation Matrix for PCP, Panel, Clinic, and Team Collaboration Factors **eAppendix 4.** Ordinary Least Squares Regression Models Depicting Adjusted Associations of PCP, Panel, Clinic, and Team Collaboration Factors With Total EHR Time Per Visit, Total Pajama Time Per Visit, and Total Electronic Inbox Time Per Visit **eAppendix 5.** Generalized Estimating Equation Models Depicting Adjusted Associations of PCP, Panel, Clinic, and Team Collaboration Factors With Total EHR Time Per Visit, Total Pajama Time Per Visit, and Total Electronic Inbox Time Per Visit With Covariates in Quartiles **eAppendix 6.** Generalized Estimating Equation Models Depicting Adjusted Associations of PCP, Panel, Clinic, and Team Collaboration Factors With Total EHR Time Per Visit, Total Pajama Time Per Visit, and Total Electronic Inbox Time Per Visit With Continuous Covariates

Supplement 2.
Data Sharing Statement
