# Supplementary material for: System-Level Factors and Time Spent on Electronic Health Records by Primary Care Physicians
Source: JAMA Netw Open. 2023 Nov 22;6(11):e2344713. doi: 10.1001/jamanetworkopen.2023.44713 (PMC10665969; doi:10.1001/jamanetworkopen.2023.44713)
Supplement: Supplement 1. — eAppendix 1. Flow Diagram of PCP Inclusion in Study Sample eAppendix 2. PCP and Clinic Variation in Time Outside of Scheduled Hours Per Visit and Factors Associated with Variation in Time Outside of Scheduled Hours Per Visit in Multivariable Generalized Estimating Equation Model eAppendix 3. Correlation Matrix for PCP, Panel, Clinic, and Team Collaboration Factors eAppendix 4. Ordinary Least Squares Regression Models Depicting Adjusted Associations of PCP, Panel, Clinic, and Team Collaboration Factors With Total EHR Time Per Visit, Total Pajama Time Per Visit, and Total Electronic Inbox Time Per Visit eAppendix 5. Generalized Estimating Equation Models Depicting Adjusted Associations of PCP, Panel, Clinic, and Team Collaboration Factors With Total EHR Time Per Visit, Total Pajama Time Per Visit, and Total Electronic Inbox Time Per Visit With Covariates in Quartiles eAppendix 6. Generalized Estimating Equation Models Depicting Adjusted Associations of PCP, Panel, Clinic, and Team Collaboration Factors With Total EHR Time Per Visit, Total Pajama Time Per Visit, and Total Electronic Inbox Time Per Visit With Continuous Covariates [file jamanetwopen-e2344713-s001.pdf]

Supplemental Online Content

Rotenstein LS, Holmgren AJ, Horn DM, et al. System-level factors and time spent on the electronic health record by primary care physicians. *JAMA Netw Open*. 2023;6(11):e2344713. doi:10.1001/jamanetworkopen.2023.44713

eAppendix 1. Flow Diagram of PCP Inclusion in Study Sample

eAppendix 2. PCP and Clinic Variation in Time Outside of Scheduled Hours Per Visit and Factors Associated with Variation in Time Outside of Scheduled Hours Per Visit in Multivariable Generalized Estimating Equation Model

eAppendix 3. Correlation Matrix for PCP, Panel, Clinic, and Team Collaboration Factors

Appendix 4. Ordinary Least Squares Regression Models Depicting Adjusted Associations of PCP, Panel, Clinic, and Team Collaboration Factors with Total EHR Time Per Visit, Total Pajama Time Per Visit, and Total Electronic Inbox Time Per Visit

Appendix 5. Generalized Estimating Equation Models Depicting Adjusted Associations of PCP, Panel, Clinic, and Team Collaboration Factors with Total EHR Time Per Visit, Total Pajama Time Per Visit, and Total Electronic Inbox Time Per Visit With Covariates in Quartiles

Appendix 6. Generalized Estimating Equation Models Depicting Adjusted Associations of PCP, Panel, Clinic, and Team Collaboration Factors With Total EHR Time Per Visit, Total Pajama Time Per Visit, and Total Electronic Inbox Time Per Visit With Continuous Covariates

This supplemental material has been provided by the authors to give readers additional information about their work.

© 2023 Rotenstein LS et al. *JAMA Network Open.*

Appendix 1. Flow Diagram of PCP Inclusion in Study Sample

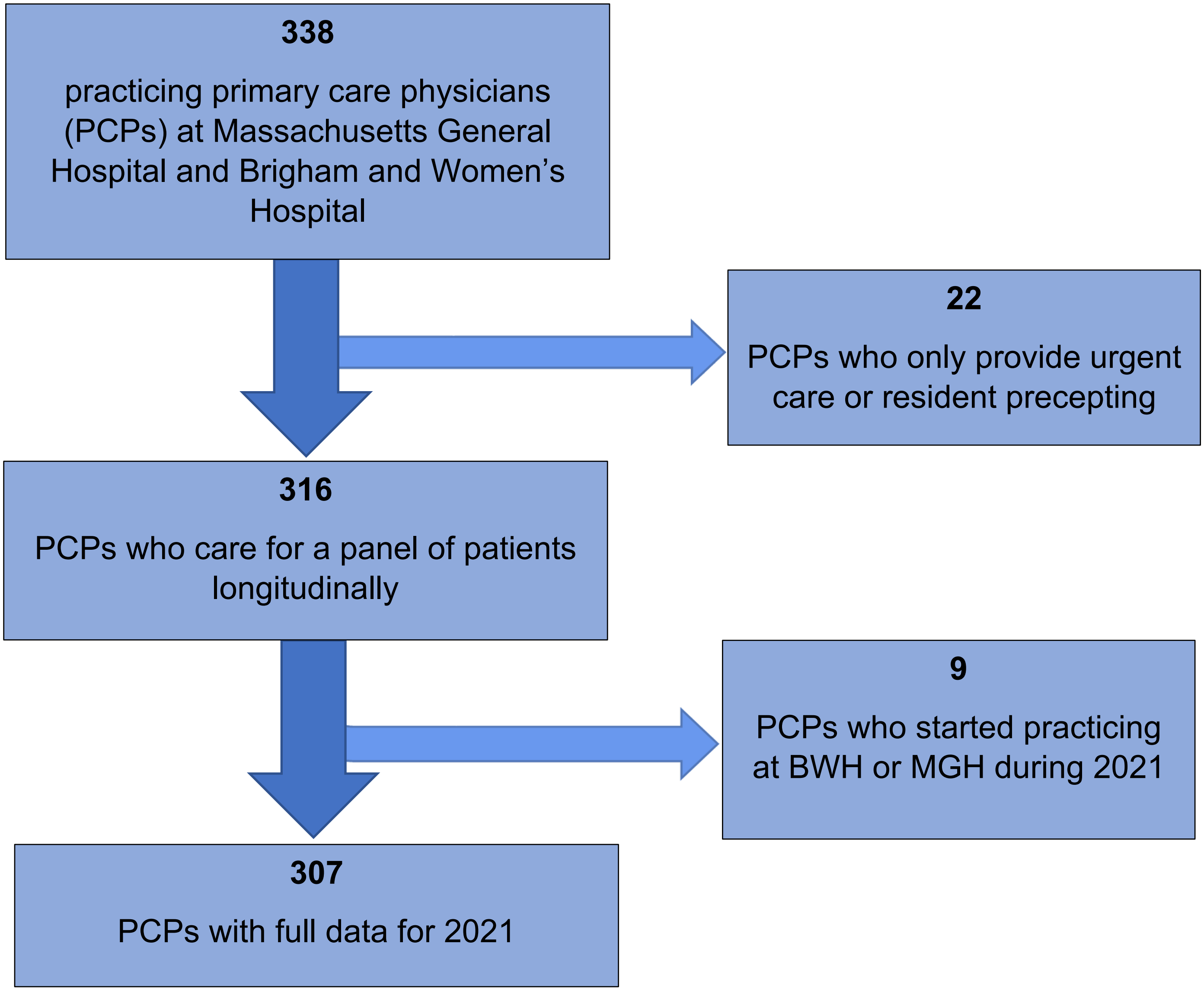

© 2023 Rotenstein LS et al. *JAMA Network Open.*

eAppendix 2. PCP and Clinic Variation in Time Outside of Scheduled Hours Per Visit and Factors Associated with Variation in Time Outside of Scheduled Hours Per Visit in Multivariable Generalized Estimating Equation Model

a. PCP Variation

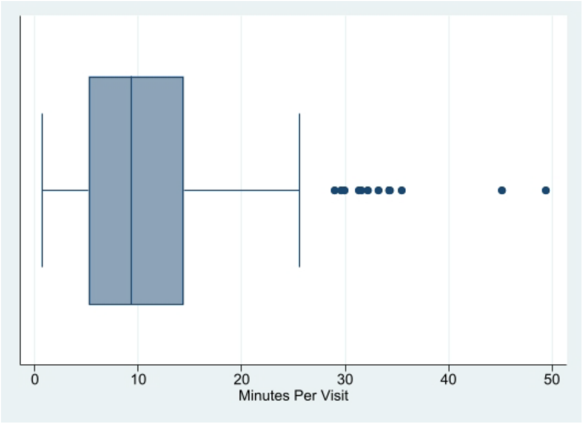

b. Clinic Variation

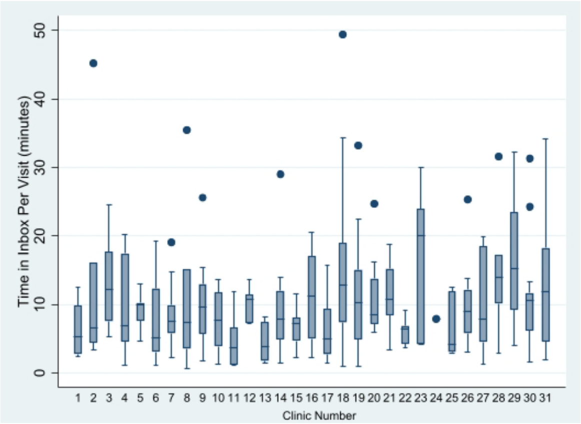

c. Factors Associated with Variation in Time Outside of Scheduled Hours Per Visit

	Total Time Outside of Scheduled Hours Per Visit	
**Parameter**	**Estimate (95% CI), min/day**	**P-Value**
**PCP Factors**		
**PCP Gender** (Female vs. Male)	2.78 (0.25, 5.31)	0.03
**Clinical FTE**	0.04 (-8.70, 8.78)	0.99
**Number of Years Post-Residency**	0.11 (0.04, 0.18)	0.001
**Patient Panel Factors**		
**Above Median Percent Female Patients on Panel (Yes vs. No)**	-1.87 (-4.35, 0.60)	0.14
**Above Median Percent Patients with Medicaid on Panel (Yes vs. No)**	0.49 (-2.41, 3.40)	0.74
**Above Median Panel Risk Score (Yes vs. No)**	-1.80 (-4.24, 0.63)	0.15
**Primary Care Clinic Factors**		
**MA FTE to Provider FTE Ratio Above Median (Yes vs. No)**	-0.80 (-2.57, 0.96)	0.37
**Nurse FTE to Provider FTE Ratio Above Median (Yes vs. No)**	-0.82 (-2.36, 0.72)	0.30
**Secretarial Staff FTE to Provider FTE Ratio Above Median (Yes vs. No)**	1.06 (-0.66, 2.79)	0.23
**Presence of a Pharmacy Technician (Yes vs. No)**	-3.03 (-6.50, 0.44)	0.09
**Community Health Center (Yes vs. No)**	-3.04 (-6.52, 0.45)	0.09
**Team Collaboration**		
**Use of a Scribe (Yes vs. No)**	0.41 (-1.28, 2.10)	0.63
**Above Median Team Contribution to Orders (Yes vs. No)**	-1.55 (-3.56, 0.45)	0.13

*Models additionally control for institution (BWH vs. MGH), panel size (number of patients), and yearly message quantity (number of messages).

© 2023 Rotenstein LS et al. *JAMA Network Open.*

eAppendix 3. Correlation Matrix for PCP, Panel, Clinic, and Team Collaboration Factors

	Avg Total EHR Time Per Visit	Avg Pajama Time Per Visit	Avg Electronic Inbox Time Per Visit	Avg Time Outside Scheduled	Clinical FTE	Scribe	Years Post Residency	Pct of Team Contribution to Orders	Pct Female Patients on Panel	Pct of Patients with Medicaid on Panel	Average Panel Risk Score	Panel Size	Total Yearly Messages	MA to Provider Ratio	Nurse to Provider Ratio	Secretarial Staff to Provider Ratio	Pharm Tech FTEs
**Average Total EHR Time Per Visit**	1.000	0.823	0.811	0.821	-0.325	-0.071	0.083	-0.116	0.069	-0.009	0.195	-0.349	-0.140	-0.067	-0.070	0.060	-0.005
**Average Pajama Time Per Visit**	0.823	1.000	0.696	0.640	-0.198	-0.019	0.172	-0.062	0.030	-0.054	0.149	-0.203	-0.013	0.012	-0.081	0.065	-0.015
**Average Electronic Inbox Time Per Visit**	0.811	0.696	1.000	0.660	-0.173	0.014	0.144	-0.046	0.073	-0.023	0.162	-0.226	0.015	0.002	-0.042	0.091	-0.024
**Average Time Outside Scheduled Hours Per Visit**	0.821	0.640	0.660	1.000	-0.399	0.028	-0.003	-0.078	0.093	-0.004	0.250	-0.397	-0.092	-0.078	-0.043	0.045	-0.064
**Clinical FTE**	-0.325	-0.198	-0.173	-0.399	1.000	0.138	0.030	0.068	-0.033	-0.066	-0.129	0.845	0.745	0.231	0.118	-0.097	-0.335
**Scribe**	-0.071	-0.019	0.014	0.028	0.138	1.000	-0.059	0.186	0.037	0.190	0.298	0.004	0.165	-0.184	0.032	-0.247	-0.340
**Years Post Residency**	0.083	0.172	0.144	-0.003	0.030	-0.059	1.000	0.199	-0.030	-0.227	0.265	-0.002	0.052	-0.047	0.022	-0.005	-0.092
**Percent of Team Contribution to Orders**	-0.116	-0.062	-0.046	-0.078	0.068	0.186	0.199	1.000	0.097	-0.142	0.229	-0.020	0.080	-0.048	0.058	-0.027	-0.182
**Percent Female Patients on Panel**	0.069	0.030	0.073	0.093	-0.033	0.037	-0.030	0.097	1.000	0.001	-0.017	-0.008	0.097	0.029	0.031	0.021	0.014
**Percent of Patients with Medicaid on Panel**	-0.009	-0.054	-0.023	-0.004	-0.066	0.190	-0.227	-0.142	0.001	1.000	-0.110	-0.174	-0.013	-0.042	0.384	0.131	-0.018
**Average Panel Risk Score**	0.195	0.149	0.162	0.250	-0.129	0.298	0.265	0.229	-0.017	-0.110	1.000	-0.344	0.025	-0.367	-0.001	-0.145	-0.313
**Panel Size**	-0.349	-0.203	-0.226	-0.397	0.845	0.004	-0.002	-0.020	-0.008	-0.174	-0.344	1.000	0.729	0.326	-0.006	-0.054	-0.245
**Total Yearly Messages**	-0.140	-0.013	0.015	-0.092	0.745	0.165	0.052	0.080	0.097	-0.013	0.025	0.729	1.000	0.219	0.084	-0.011	-0.347
**MA to Provider Ratio**	-0.067	0.012	0.002	-0.078	0.231	-0.184	-0.047	-0.048	0.029	-0.042	-0.367	0.326	0.219	1.000	-0.001	0.386	-0.156
**Nurse to Provider Ratio**	-0.070	-0.081	-0.042	-0.043	0.118	0.032	0.022	0.058	0.031	0.384	-0.001	-0.006	0.084	-0.001	1.000	0.060	-0.346
**Secretarial Staff to Provider Ratio**	0.060	0.065	0.091	0.045	-0.097	-0.247	-0.005	-0.027	0.021	0.131	-0.145	-0.054	-0.011	0.386	0.060	1.000	0.206
**Pharmacy Technician FTEs**	-0.005	-0.015	-0.024	-0.064	-0.335	-0.340	-0.092	-0.182	0.014	-0.018	-0.313	-0.245	-0.347	-0.156	-0.346	0.206	1.000

© 2023 Rotenstein LS et al. *JAMA Network Open.*

Appendix 4. Ordinary Least Squares Regression Models Depicting Adjusted* Associations of PCP, Panel, Clinic, and Team Collaboration Factors with Total EHR Time Per Visit, Total Pajama Time Per Visit, and Total Electronic Inbox Time Per Visit

	Total EHR Time Per Visit $R^2 = 0.23$			Total Pajama Time Per Visit $R^2 = 0.17$			Total Inbox Time Per Visit $R^2 = 0.20$		
**Parameter**	**Estimate (95% CI), min/day**	**P-Value**	**VIF**	**Estimate (95% CI), min/day**	**P-Value**	**VIF**	**Estimate (95% CI), min/day**	**P-Value**	**VIF**
**PCP Factors**									
**PCP Gender** (Female vs. Male)	4.20 (-2.42, 10.83)	0.21	2.89	2.56 (-0.94, 6.05)	0.15	2.88	1.03 (-0.98, 3.05)	0.30	2.89
**Clinical FTE**	-8.35 (-25.47, 8.77)	0.33	4.85	-5.50 (-14.23, 3.22)	0.21	4.81	-6.40 (-9.09, -3.70)	<0.001	4.85
**Number of Years Post-Residency**	0.13 (0.00, 0.26)	0.05	1.21	0.15 (0.07, 0.24)	0.001	1.21	0.00 (-0.04, 0.03)	0.95	1.21
**Patient Panel Factors**									
**Above Median Percent Female Patients on Panel (Yes vs. No)**	-1.55 (-8.31, 5.20)	0.64	2.82	-2.43 (-6.34, 1.47)	0.21	2.81	-0.34 (-2.10, 1.42)	0.70	2.82
**Above Median Percent Patients with Medicaid on Panel (Yes vs. No)**	1.64 (-2.12, 5.40)	0.38	1.81	-0.28 (-2.55, 1.99)	0.80	1.81	-0.01 (-0.73, 0.71)	0.98	1.81
**Above Median Panel Risk Score (Yes vs. No)**	-2.24 (-6.35, 1.86)	0.27	2.59	-1.80 (-4.03, 0.43)	0.11	2.58	-0.24 (-1.34, 0.86)	0.66	2.59
**Primary Care Clinic Factors**									
**MA FTE to Provider FTE Ratio Above Median (Yes vs. No)**	-2.80 (-6.52, 0.93)	0.14	1.46	-1.02 (-2.91, 0.88)	0.28	1.45	0.33 (-0.40, 1.06)	0.37	1.46
**Nurse FTE to Provider FTE Ratio Above Median (Yes vs. No)**	-1.74 (-4.94, 1.45)	0.27	1.45	-1.24 (-2.75, 0.27)	0.10	1.45	-0.09 (-0.84, 0.66)	0.81	1.45
**Secretarial Staff FTE to Provider FTE Ratio Above Median (Yes vs. No)**	1.76 (-1.62, 5.14)	0.30	1.39	1.65 (-0.18, 3.48)	0.08	1.39	0.01 (-0.79, 0.81)	0.98	1.39
**Presence of a Pharmacy Technician (Yes vs. No)**	-7.87 (-14.23, -1.52)	0.02	2.81	-3.73 (-7.59, 0.13)	0.06	2.78	-1.65 (-3.17, -0.12)	0.04	2.81
**Community Health Center (Yes vs. No)**	-5.40 (-11.21, 0.41)	0.07	2.16	-1.97 (-4.54, 0.61)	0.13	2.16	-1.68 (-3.06, -0.29)	0.02	2.16
**Team Collaboration**									
**Use of a Scribe (Yes vs. No)**	-3.32 (-7.03, 0.39)	0.08	1.50	-0.09 (-2.18,1.99)	0.93	1.50	-0.11 (-1.13, 0.91)	0.82	1.50
**Above Median Team Contribution to Orders (Yes vs. No)**	-3.81 (-7.42, -0.20)	0.04	1.34	-2.55 (-4.57, -0.52)	0.02	1.33	-1.48 (-2.21, -0.74)	0.0003	1.34

*Models additionally control for institution (BWH vs. MGH), panel size (number of patients), and yearly message quantity (number of messages).

© 2023 Rotenstein LS et al. *JAMA Network Open.*

Appendix 5. Generalized Estimating Equation Models Depicting Adjusted* Associations of PCP, Panel, Clinic, and Team Collaboration Factors with Total EHR Time Per Visit, Total Pajama Time Per Visit, and Total Electronic Inbox Time Per Visit with Covariates in Quartiles

		Total EHR Time Per Visit		Total Pajama Time Per Visit		Total Inbox Time Per Visit	
**Parameter**		**Estimate (95% CI), min/day**	**P-Value**	**Estimate (95% CI), min/day**	**P-Value**	**Estimate (95% CI), min/day**	**P-Value**
**PCP Factors**							
**PCP Gender (Female vs. Male)**		5.33 (-3.18, 13.83)	0.22	2.60 (-1.93, 7.13)	0.26	1.24 (-0.97, 3.45)	0.27
**Clinical FTE**		-9.81 (-28.67, 9.06)	0.31	-6.93 (-17.06, 3.20)	0.18	-6.91 (-10.09, -3.72)	<0.001
**Years Post-Residency**		0.14 (0.01, 0.27)	0.03	0.14 (0.07, 0.22)	0.0001	0.00 (-0.04,0.04)	0.95
**Patient Panel Factors**							
**Percent of Female Patients on Panel Quartile**	2 vs. 1	-2.27 (-8.94, 4.39)	0.50	-0.54 (-3.32, 2.24)	0.70	-0.33 (-1.59, 0.94)	0.61
	3 vs. 1	-3.23 (-14.80, 8.35)	0.58	-2.52 (-8.64, 3.61)	0.42	-0.43 (-2.81, 1.95)	0.72
	4 vs. 1	-3.39 (-13.59, 6.80)	0.51	-2.23 (-7.94, 3.48)	0.44	-0.90 (-3.39, 1.60)	0.48
**Percent Patients with Medicaid on Panel Quartile**	2 vs. 1	-4.55 (-8.64, -0.46)	0.03	-1.24 (-4.02, 1.54)	0.38	-1.47 (-2.43, -0.50)	0.003
	3 vs. 1	0.13 (-4.77, 5.04)	0.96	0.19 (-3.00, 3.37)	0.91	-0.85 (-1.80, 0.09)	0.08
	4 vs. 1	6.21 (-0.01, 12.42)	0.05	0.65 (-2.61, 3.91)	0.70	0.12 (-1.28, 1.53)	0.86
**Panel Risk Score Quartile**	2 vs. 1	-0.69 (-4.23, 2.86)	0.70	-0.83 (-3.03, 1.36)	0.46	-0.27 (-1.35, 0.81)	0.63
	3 vs. 1	-4.48 (-9.89, 0.94)	0.11	-3.18 (-6.26, -0.10)	0.04	-0.56 (-1.98, 0.86)	0.44
	4 vs. 1	0.50 (-8.70, 9.70)	0.92	-0.24 (-5.37, 4.89)	0.93	-0.14 (-2.50, 2.23)	0.91
**Primary Care Clinic Factors**							
**MA FTE to Provider FTE Ratio Quartile**	2 vs. 1	0.96 (-2.64 4.57)	0.60	2.15 (-0.22, 4.52)	0.08	-0.80 (-1.73, 0.13)	0.09
	3 vs. 1	-5.46 (-9.88 -1.04)	0.02	-2.62 (-5.75, 0.51)	0.10	-0.96 (-2.22, 0.29)	0.13
	4 vs. 1	0.26 (-3.83, 4.36)	0.90	0.95 (-1.27, 3.17)	0.40	0.69 (-0.26, 1.64)	0.16
**Nurse FTE to Provider FTE Ratio Quartile**	2 vs. 1	2.85 (-1.96, 7.67)	0.25	1.02 (-1.64, 3.69)	0.45	0.54 (-0.65, 1.72)	0.37
	3 vs. 1	-0.92 (-5.23, 3.40	0.68	-1.63 (-4.32, 1.05)	0.23	0.35 (-0.77, 1.47)	0.54
	4 vs. 1	-0.48 (-4.70, 3.73)	0.82	-1.55 (-3.78, 0.69)	0.17	0.69 (-0.45, 1.84)	0.24
**Secretarial Staff FTE to Provider FTE Ratio Quartile**	2 vs. 1	-1.05 (-6.14, 4.05)	0.69	-0.02 (-3.45, 3.41)	0.99	0.60 (-0.84, 2.04)	0.41
	3 vs. 1	-2.63 (-7.15, 1.90)	0.26	-1.79 (-4.45, 0.87)	0.19	-0.02 (-1.08, 1.03)	0.97
	4 vs. 1	2.56 (-1.24, 6.37)	0.19	1.58 (-0.79, 3.96)	0.19	0.57 (-0.33, 1.46)	0.21
**Presence of a Pharmacy Tech (Yes vs. No)**		-6.20 (-11.56, -0.84)	0.02	-1.94 (-5.36, 1.48)	0.27	-1.74 (-3.10, -0.37)	0.01
**Community Health Center (Yes vs. No)**		-9.50 (-15.10, -3.90	0.0009	-2.30 (-5.62, 1.02)	0.18	-2.32 (-3.91, -0.73)	0.004
**Team Collaboration**							
**Use of a Scribe (Yes vs. No)**		-3.78 (-7.53, -0.02)	0.05	-0.39 (-2.51, 1.73)	0.72	-0.17 (-1.10, 0.75)	0.71
**Team Contribution to Orders Quartile**	2 vs. 1	-3.80 (-7.92, 0.32)	0.07	-2.58 (-5.61, 0.44)	0.09	-0.42 (-1.26, 0.41)	0.32
	3 vs. 1	-6.43 (-10.68, -2.18)	0.003	-5.30 (-7.89, -2.71)	<0.001	-1.55 (-2.31, -0.79)	<0.001
	4 vs. 1	-6.87 (-10.85, -2.89)	0.0007	-3.96 (-7.08, -0.85)	0.01	-1.62 (-2.54, -0.69)	0.0006

*Models additionally control for institution (BWH vs. MGH), panel size (number of patients), and yearly message quantity (number of messages).

© 2023 Rotenstein LS et al. *JAMA Network Open.*

Appendix 6. Generalized Estimating Equation Models Depicting Adjusted* Associations of PCP, Panel, Clinic, and Team Collaboration Factors with Total EHR Time Per Visit, Total Pajama Time Per Visit, and Total Electronic Inbox Time Per Visit with Continuous Covariates

	Total EHR Time Per Visit		Total Pajama Time Per Visit		Total Inbox Time Per Visit	
**Parameter**	**Estimate (95% CI), min/day**	**P-Value**	**Estimate (95% CI), min/day**	**P-Value**	**Estimate (95% CI), min/day**	**P-Value**
**PCP Factors**						
**PCP Gender** (Female vs. Male)	5.56 (-4.41, 15.53)	0.27	1.89 (-4.14, 7.91)	0.54	1.38 (-1.14, 3.90)	0.284
**Clinical FTE**	-7.74 (-22.82, 7.33)	0.31	-5.51 (-12.70, 1.68)	0.13	-6.84 (-9.46, -4.22)	< 0.001
**Years Post-Residency**	0.18 (0.05, 0.31)	0.006	0.16 (0.08, 0.24)	< 0.001	0.01 (-0.03, 0.04)	0.68
**Patient Panel Factors**						
**Percent of Female Patients on Panel**	-7.66 (-30.93, 15.62)	0.52	-3.79 (-18.15, 10.57)	0.61	-2.04 (-7.20, 3.13)	0.44
**Percent of Patients with Medicaid on Panel**	0.13 (-0.08, 0.33)	0.22	-0.01 (-0.10, 0.07)	0.74	-0.01 (-0.06, 0.04)	0.71
**Panel Risk Score**	0.16 (-6.63, 6.95)	0.96	-0.54 (-4.78, 3.70)	0.80	-0.52 (-2.28, 1.24)	0.56
**Primary Care Clinic Factors**						
**MA FTE to Provider FTE Ratio**	3.40 (-4.52, 11.32)	0.40	3.20 (-0.92, 7.32)	0.13	1.54 (-0.35, 3.43)	0.11
**Nurse FTE to Provider FTE Ratio**	-2.74 (-13.21, 7.72)	0.61	-3.41 (-8.03, 1.21)	0.15	0.65 (-2.64, 3.95)	0.70
**Secretarial Staff FTE to Provider FTE Ratio**	1.04 (-6.85, 8.93)	0.80	1.05 (-3.26, 5.37)	0.63	0.18 (-1.79, 2.14)	0.86
**Presence of a Pharmacy Technician**	-6.96 (-12.67, -1.24)	0.02	-2.65 (-5.94, 0.64)	0.11	-1.34 (-2.61, -0.06)	0.04
**Community Health Center**	-8.82 (-16.83, -0.81)	0.03	-1.80 (-5.48, 1.87)	0.34	-1.58 (-3.44, 0.27)	0.09
**Team Collaboration**						
**Use of a Scribe**	-2.29 (-5.82, 1.25)	0.20	0.31 (-1.73, 2.36)	0.76	-0.09 (-0.84, 1.01)	0.86
**Percent of Orders with Team Contribution**	-0.40 (-0.53, -0.28)	< 0.001	-0.17 (-0.25, -0.09)	< 0.001	-0.09 (-0.13, -0.05)	< 0.001

*Models additionally control for institution (BWH vs. MGH), panel size (number of patients), and yearly message quantity (number of messages).

© 2023 Rotenstein LS et al. *JAMA Network Open.*
