# Supplementary material for: System-Level Factors and Time Spent on Electronic Health Records by Primary Care Physicians
Source: JAMA Netw Open. 2023 Nov 22;6(11):e2344713. doi: 10.1001/jamanetworkopen.2023.44713 (PMC10665969; doi:10.1001/jamanetworkopen.2023.44713)
Supplement: Supplement 2. — Data Sharing Statement [file jamanetwopen-e2344713-s002.pdf]

Data Sharing Statement

Rotenstein. System-Level Factors and Time Spent on Electronic Health Records by Primary Care Physicians. *JAMA Netw Open*. Published November 22, 2023. doi:10.1001/jamanetworkopen.2023.44713

Data
Data available: No
